# Scalable Accelerated Materials Discovery of Sustainable Polysaccharide-Based Hydrogels by Autonomous Experimentation and Collaborative Learning

**DOI:** 10.1021/acsami.4c16614

**Published:** 2024-12-11

**Authors:** Yang Liu, Xubo Yue, Junru Zhang, Zhenghao Zhai, Ali Moammeri, Kevin J. Edgar, Albert S. Berahas, Raed Al Kontar, Blake N. Johnson

**Affiliations:** † Grado Department of Industrial and Systems Engineering, Virginia Tech, Blacksburg, Virginia 24061, United States; ‡ Macromolecules Innovation Institute, Virginia Tech, Blacksburg, Virginia 24061, United States; § Department of Mechanical and Industrial Engineering, Northeastern University, Boston, Massachusetts 02115, United States; ∥ Department of Chemical Engineering, Virginia Tech, Blacksburg, Virginia 24061, United States; ⊥ Department of Sustainable Biomaterials, Virginia Tech, Blacksburg, Virginia 24061, United States; # Department of Industrial and Operations Engineering, University of Michigan, Ann Arbor, Michigan 48109, United States; ∇ Department of Materials Science and Engineering, Virginia Tech, Blacksburg, Virginia 24061, United States

**Keywords:** materials genome initiative, autonomous experimentation, Bayesian optimization, active learning, glycomaterials

## Abstract

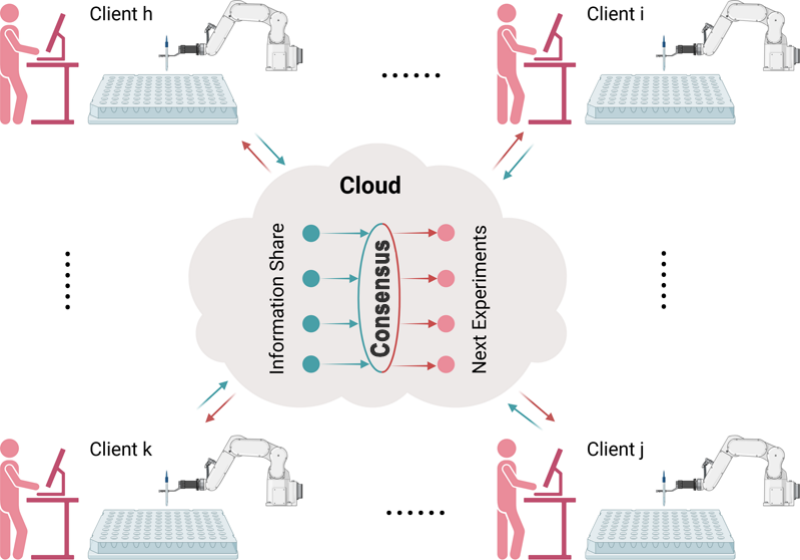

While some materials can be discovered and engineered using standalone self-driving workflows, coordinating multiple stakeholders and workflows toward a common goal could advance autonomous experimentation (AE) for accelerated materials discovery (AMD). Here, we describe a scalable AMD paradigm based on AE and “collaborative learning”. Collaborative learning using a novel consensus Bayesian optimization (BO) model enabled the rapid discovery of mechanically optimized composite polysaccharide hydrogels. The collaborative workflow outperformed a non-collaborating AMD workflow scaled by independent learning based on the trend of mechanical property evolution over eight experimental iterations, corresponding to a budget limit. After five iterations, four collaborating clients obtained notable material performance (i.e., composition discovery). Collaborative learning by consensus BO can enable scaling and performance optimization for a range of self-driving materials research workflows driven by optimally cooperating humans and machines that share a material design objective.

## 1 Introduction

The Materials Genome Initiative (MGI) aims to achieve rapid and cost-effective materials discovery and engineering. Hybrid materials research infrastructures, including integrated tools, methods, and processes for experimentation, computation, and data analytics, are needed to achieve this goal.^1,2^ Automation and machine learning have significantly advanced experimental resources for accelerated materials discovery (AMD), such as establishing workflows based on high-throughput experimentation (HTE)^3,4^ and autonomous experimentation (AE).^5,6^ While AMD can be achieved by brute force (e.g., HTE) in applications that exhibit a simple design space or large budget,^7^ workflow performance and discovery outcomes can be transformed by integrating automation and adaptive sampling methods, such as for AE, in applications that exhibit budget constraints. Given the complexity of functional material composition–process–structure–property relations (i.e., design spaces), adaptive sampling strategies have shown promise to improve several workflow performance metrics, including speed and efficiency, thereby enabling AMD in applications that may exhibit severe budget and resource constraints.^8,9^ In particular, applications to AMD of soft materials (e.g., hydrogels) for biomedical applications can exhibit complex design spaces and high experimental cost. While HTE and AMD application to hydrogels is an emerging area (see Table S1),^10−13^ applications focused on hard materials have shown value of integrating experimental and computation tools.^2,14^

Bayesian optimization (BO) and active learning use adaptive sampling to achieve a specific learning goal.^15^ Active learning, sometimes called “query learning”, is the study of machine learning systems that improve by asking questions.^16^ Active learning is instrumental when unlabeled data are numerous, labeling consumes significant resources (e.g., physical or economic), or it is anticipated that many data must be labeled to train a model.^16^ There are many ways by which a learner can ask queries. Kusne et al. used Bayesian active learning to discover phase-change memory materials by identifying the composition-phase structure relation over 19 experimental iterations.^17^ Min et al. used active learning to discover inorganic materials based on efficient global optimization that satisfy band gap and refractive index constraints over 50 experimental iterations.^18^ Oftelie et al. used active learning to design layered materials using BO by identifying optimal structures for band gap and electronic fitness function values.^19^

BO is a sequential process that aims to reduce the number of required experiments by optimizing an unknown black-box (BB) function,^20^ such as the true relationship between a material’s composition and properties. BO begins by using a relatively small number of initial data points to learn a surrogate model, often a Gaussian Process (GP) given its intrinsic ability to quantify uncertainty,^21^ that approximates the BB function.^22^ Subsequently, BO relies on a utility function based on the surrogate to quantify the potential benefits of conducting new experiments (e.g., testing a new formulation). By optimizing the utility function, which balances exploration and exploitation, one can select a new formulation for analysis that will best yield the desired yet unknown composition–process–structure–property relation. Exploration refers to allowing for new feature discovery, while exploitation refers to capitalizing on knowledge already gained. These newly labeled data are then integrated with the existing data set, and the cycle is iterated until available resources (e.g., budget) are utilized or an exit condition is met. Burger et al. implemented BO on a robot to autonomously search for photocatalysts with improved hydrogen production performance.^23^ Shields et al. used BO to identify the top-yielding conditions in the Mitsunobu reaction and deoxyfluorination reaction processes.^24^

While adaptive sampling can improve the performance of AMD workflows, such as allowing them to operate under budget constraints, it is prudent to consider its impact on workflow scalability. For example, it may be desirable for multiple clients to leverage their workflows toward a common AMD objective, such as by hierarchical modeling.^25^ Advances in the computation and communication power of edge devices^26^ now make it plausible for multiple clients, such as humans and machines (e.g., HTE or AE systems), to share information, distribute trial-and-error efforts, and fast-track experimental processes such that all participants benefit, which is a crucial aspect of AMD scalability and performance optimization. Unfortunately, despite its utility in AE and AMD, conventional adaptive sampling methods lack a collaborative element. Thus, there remains a critical need to integrate principles of collaboration with adaptive sampling methods to scale HTE and AE, and AMD applications thereof, using networks of distributed humans and machines.

Here, we demonstrate a methodology for scalable self-driving AMD founded on AE and collaborative learning. We demonstrate the utility and impact of a novel consensus BO framework in which multiple collaborating “clients” locally perform experiments and agree (i.e., reach a consensus) on their individual “next-to-test” formulations to achieve a shared material design objective. This work showcases the potential of merging AE and collaborative machine learning to expedite and scale material discovery initiatives subject to resource and privacy constraints (e.g., budget).

## 2 Materials and Methods

### 2.1 Collaborative Learning via Consensus BO

#### 2.1.1 Mathematical Notation

First, we define the mathematical notation used in this work. Assume there are *K* clients (e.g., experimenters, such as chemists, HTE systems, or AE systems), and each client has a budget of *T* experiments imposed by resource limitations. Let *t* ∈ {0,1,···,*T* – 1} be the iteration index. Consider  the initial data set for client *k* with *N*_*k*_^(0)^ samples, where *X*_*k*_^(0)^ = (*x*_*k*,1_,···,*x*_*k*,*N*_*k*_^(0)^_) is a matrix that contains the initial input vector data *x*_*k*_, (i.e., material composition, specifically the concentration of each component in the mixture) and *y*_*k*_^(0)^ = (*y*_*k*,1_,···,*y*_*k*,*N*_*k*_^(0)^_)^T^ is a vector that contains the corresponding outputs (i.e., numeric measures of the material properties for each composition, such as the shear storage modulus (*G*′)). Here, the input data *x*_*k*_, is of dimension *D*, where each dimension denotes a component in the material (i.e., *D* = the number of different species in the mixture). Each client’s goal is to find a formulation (i.e., mixture composition) that optimizes a target material performance or quality measure, specifically *G*′ in this work. This problem is analogous to a team of cooks attempting to discover a recipe that generates optimal taste. Mathematically, this translates to the following optimization problem:
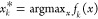
1where *f*_*k*_ is the true BB function each client aims to optimize. To observe *f*_*k*_(*x*) using a new formulation *x*, we need to conduct an experiment and observe a potentially noisy outcome *y*_*k*_(*x*). In other words, *y*_*k*_ = *f*_*k*_ + ε_*k*_, where ε_*k*_ is an additive noise.

#### 2.1.2 Independent Learning via Traditional BO

In an environment where clients operate independently, they select successive formulations to test by maximizing utility without communicating with others. More specifically, from a mathematical perspective, at each iteration *t* (i.e., experiment number), a GP surrogate model *f̂*_*k*_ is fit to the data  to estimate *f̂*_*k*_(*x*) for any input *x*. While several kernel functions are applicable for the GP model, the squared exponential kernel function was selected in this study because it defines a smooth function. Importantly, a GP provides a predictive distribution  over *f̂*_*k*_(*x*). Equipped with the predictive distribution, the *k*^th^ client then chooses the next formulation to test by maximizing their expected utility
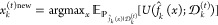
where  is a utility function that quantifies the benefit gained if one were to conduct an experiment using a new formulation *x*, and *x*_*k*_^(*t*)new^ is the recommended next-to-test formulation. Many utility functions have been developed. Among the most used is the expected improvement (EI) utility,^27^ which is defined as:
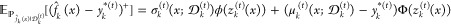
2where *a*^+^ = max(*a*,0), *y*_k_^*(*t*)^ is the current best-observed output for client *k*, ϕ (or Φ) is a probability density function (or cumulative distribution function) of a standard normal random variable, ,  is the predictive mean of the GP at input *x*, and  is the corresponding variance. The uncertainty from GP prediction (i.e., the variance information σ_*k*_^(*t*)^) enables the utility function to balance exploration and exploitation. EI can be interpreted as finding a new formulation *x* that has the potential to improve upon the current best-observed output. Surrogate modeling and utility optimization can be done using the GPytorch library.^28^

#### 2.1.3 Collaborative Learning via Consensus BO—Model Description

To foster collaboration and efficiently distribute the workload across multiple clients, we propose a “collaborative BO” approach, where cooperating clients reach a consensus on their next-to-test formulations. Through consensus, clients may borrow information from each other to conduct more efficient and effective tests (e.g., experiments). Our consensus BO approach is accomplished through a consensus matrix *W*^(*t*)^, which regulates the extent to which one client’s decision influences the choices of others. From a mathematical perspective, the new objective function is:
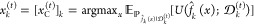
3and
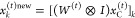
4where [·]_*k*_ represents the *k*-th block of a vector, *x*_C_^(*t*)^ is a vector that concatenates all *x*_*k*_^(*t*)^ for *k* = {1,···,*K*}, *W*^(*t*)^ is a dynamic consensus matrix, ⊗ is a Kronecker product operation, and *I* is an identity matrix. A key property of *W*^(*t*)^ is that it is a symmetric and doubly stochastic matrix (i.e., ∑_*k*_*w*_*kj*_^(*t*)^ = ∑_*j*_*w*_*kj*_^(*t*)^ = 1 for all *j*, *k* ∈ {1,···,*K*}) with non-negative elements.

The consensus BO objective function was solved by the following steps:

1. At iteration *t* ∈ {0,1,···,*T* – 1}, each client *k* solves  and obtains *x*_*k*_^(*t*)^.
2. Each client *k* sends its *x*_*k*_^(*t*)^ to a central orchestrator.
3. The orchestrator concatenates the *x*_*k*_^(*t*)^ of all clients, computes (*W*^(*t*)^ ⊗ *I*)*x*_C_^(*t*)^, and then sends *x*_*k*_^(*t*)new^ to the corresponding client *k*.
4. Each client *k* then conducts a test (e.g., experiment) using the new formulation *x*_*k*_^(*t*)new^ and observes *y*_*k*_(*x*_*k*_^(*t*)new^).
5. Each client *k* then augments the data set  by (*x*_*k*_^(*t*)new^, *y*_*k*_(*x*_*k*_^(*t*)new^)) to obtain a new data set .
6. Each client *k* then updates its GP surrogate using  and starts the new iteration *t* + 1.

The consensus BO framework naturally distributes effort (e.g., experimental workload) across the clients, allowing parallel exploration and exploitation and more efficient experimental design. Additional methodological details of the consensus BO model are provided in our previous work,^29^ and our code is provided on GitHub (https://github.com/UMDataScienceLab/Consensus_Bayesian_Opt/tree/main).

A key question that remains is how to design *W*^(*t*)^. To enable effective collaboration, *W*^(*t*)^ is initialized as a uniform matrix whose entries are equal. Mathematically, this is equivalent to:
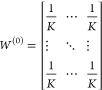
5

The dynamic consensus matrix is then gradually shrunk to an identity matrix during the collaboration process. For example, if we adjust weights linearly as:
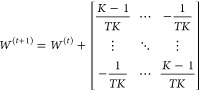
6

A finite budget *T* was assumed in eq 6. Here, *T* denotes the maximum number of experiments each client can perform using their available resources. When the budget (e.g., quantified as a number of experiments or iterations) is reached, the process stops, and the best design identified is chosen. We emphasize that reaching the budget does not imply that *W* has reached identity, which only depends on how *W* decays. Even if this occurs, our model indicates that clients should proceed using *W* = *I*, which is essentially independent BO.

An explicit boundary condition constraint can also be incorporated into the utility function (built upon the GP model) and the consensus step. First, when there is a boundary condition constraint, each local client solves the constrained BO problem
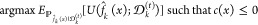
where *c*(*x*) ≤ 0 is a constraint set by the user, to ensure that the resulting *x*_*k*_^(*t*)^ lies within the boundary. The constrained function can be optimized using any BO library, such as BoTorch.^30^ Second, during the consensus step, the orchestrator concatenates all *x*_*k*_^(*t*)^ and computes (*W*^(*t*)^ ⊗ *I*)*x*_C_^(*t*)^. The resulting *x*_*k*_^(*t*)new^ for each client *k* naturally satisfies the boundary constraint since it is the convex combination of solutions {*x*_*k*_^(*t*)^}_*k*_. As for the case study, the constraints were set to be the range of the input parameters.

In summary, each client, indexed by *k*, first initially optimizes its individual utility function to determine a candidate experimental formulation, which is stored in a vector *x*_*k*_^(*t*)^. These formulation vectors are then shared with a central orchestrator and concatenated into a vector *x*_C_^(*t*)^ = (*x*_1_^(*t*)T^, ···, *x*_*K*_^(*t*)T^)^T^. Subsequently, the consensus matrix W^(*t*)^ aggregates information from all clients. This operation results in a new vector *x*^(*t*)new^ = (*x*_1_^(*t*)newT^, ···, *x*_*K*_^(*t*)newT^)^T^ that contains personalized next-to-test formulations for each client. The orchestrator then shares these next-to-test formulations with the clients for testing. This process is repeated until an exit condition is met, such as reaching a budget limit.

The consensus framework has several notable features. First, it is clear from the consensus BO objective function (i.e., eqs 3 and 4) that *W*^(*t*)^ plays a critical role in determining the next-to-test formulation for each client *k* (i.e., *x*_*k*_^(*t*)new^). *W*^(*t*)^ controls the extent to which each client affects the decisions of others. As a result, *W*^(*t*)^ adds flexibility to the optimization problem and allows a group of clients to determine the degree of collaboration dynamically (i.e., with iteration number). Second, when *W*^(*t*)^ is an identity matrix *I*, the consensus step will keep the original vector *x*_C_^(*t*)^ unchanged (i.e., (*W*^(*t*)^ ⊗ *I*)*x*_C_^(*t*)^ = (*I* ⊗ *I*)*x*_C_^(*t*)^ = *x*_C_^(*t*)^). This is equivalent to no information sharing; therefore, the consensus BO model becomes traditional BO. Third, since *W*^(*t*)^ is doubly stochastic, this ensures that the formulations of all clients will stay in the solution space that has been explored. Finally, our consensus BO objective function generates an individualized solution for each client that allows the group of clients to distribute effort (e.g., experimental or computational), allowing parallel exploration–exploitation and more efficient experimental design. The intuition behind this design is as follows. In the early stages, a given client *k* may not have enough observations to obtain a high-quality surrogate model and, therefore, needs to borrow information from other clients. In the late stages, as client *k* accumulates sufficient data and can construct a high-quality local surrogate model, it will focus more on its local problem to find a client-specific optimal formulation. This distributed active learning approach with consensus BO transforms traditional BO into a new collaborative paradigm, which can accelerate the optimal design process by effectively distributing experimental efforts across a network of clients.

### 2.2 Materials

Acetic acid (AcOH) was from Sigma-Aldrich. Multi-reducing end alginate (M-alginate) and carboxymethyl chitosan sodium salt (CMCS) were synthesized as previously reported.^31,32^ Lead zirconate titanate (PZT-5A, 72.4 × 72.4 × 0.127 mm^3^ wafer) with nickel (Ni) electrodes was from Piezo Systems (Woburn, MA). Glass cylinders and ethanol (200 proof) were from Fisher Scientific. Polyurethane (Fast-Drying) and Loctite EA 1C-LV epoxy adhesive were from Minwax and Henkel, respectively. Deionized water (DIW; 18.2 MΩ) was from a commercial system (Direct-Q 3 UV Water Purification Systems; Millipore).

### 2.3 High-Throughput Synthesis

Material libraries of unique composition that spanned the material design space, specifically the formulation space, were fabricated in a 96-well plate using a previously reported automated HTE system based on robotic dispensing and sensing.^7^ The precursor solutions were dispensed from a syringe (55 or 10 mL) using a straight cylindrical nozzle (32 or 27 gauge, respectively). Samples in the first library were formulated with 0.75 wt % M-alginate and 0.75 wt % CMCS precursor solutions using identical dispensing times. The total concentration of M-alginate and CMCS in each sample was a constant 0.66 wt % (i.e., 0.33 wt % M-alginate and 0.33 wt % CMCS). After dispensing, each well’s precursor solutions were robotically mixed for 30 s using a helical stirring tool. The mass of AcOH dispensed across all wells ranged from 0.02 to 52 mg. Varying but known quantities of DIW were then dispensed across all samples to make the total mass of each sample identical (390 mg). The composite polysaccharide hydrogels were then obtained by allowing the cross-linking reaction to proceed for 12 h in a closed, humid environment at room temperature. The experiment was thrice performed.

Samples in a second library were formulated with 0.75 wt % M-alginate and 0.75 wt % CMCS precursor solutions with dispensing time per well ranging from 0 to 23.5 s and 23.5 to 0 s, respectively, using a step size of 0.5 s. M-alginate and CMCS precursor solutions were dispensed in opposite paths. The total concentration of M-alginate and CMCS in each sample (i.e., well) ranged from 0–0.66 wt %, but the total solids concentration of each sample (i.e., the sum of the M-alginate and CMCS weight percentages) was a constant 0.66 wt % (see Supporting Data Set). After dispensing, each well’s precursor solutions were robotically mixed for 30 s using a helical stirring tool. Varying but known volumes of AcOH was then dispensed across the wells. Three concentrations of AcOH were used depending on the given library to maximize the explored design space. The dispensing time of AcOH (17.4 M) ranged from 0.8 to 10 s across the plate with a step size of 0.4 s. The dispensing time of 8.3 and 0.69 wt % AcOH solutions ranged from 0.5 to 6 s with a step size of 0.5 s. Varying but known quantities of DIW were then dispensed across all samples to make the total mass of each sample identical (390 mg). The composite polysaccharide hydrogels were formed as described for the first library. Three sample replicates were prepared and simultaneously characterized for each formulation.

### 2.4 High-Throughput Characterization

Hydrogel viscoelastic properties, specifically the shear storage modulus (*G*′), were measured using a previously reported well plate-compatible high-throughput characterization (HTC) system.^33^ The dynamic-mode cantilever rheometer fabrication method and measurement principle can be found in our previous reports.^33−36^ Measurements were obtained using a 60 s dwell time per sample to generate a steady-state sensor response. The sensor stabilization time between samples was 60 s. Following analysis of the last sample, the rheometer signal was continuously monitored to verify a stable baseline. The mechanical property of each sample, specifically *G*′, is correlated with the cantilever rheometer phase angle at resonance (φ) and was calculated based on the steady-state values of φ and resonant frequency (*f*_0_) obtained during the dwell period using the fluid–structure interaction model of Mather et al.^37^ The associated shear rate is 2π*f*_0_.

### 2.5 Scalable Self-Driving AMD via AE and Collaborative Learning

HTC of initial material libraries formulated by high-throughput synthesis identified the feasible input domain for the collaborative and non-collaborative (i.e., independent) learning models. Specifically, the labeled data generated from the initial sample library identified the feasible range for each mixture component. These labeled data, which established constraints on the design space, were also used to generate the first-to-test formulations for each client in the collaborative learning and control groups for iteration 0. Five points per client were sampled from these initial labeled data based on Latin hypercube design,^38^ which yielded the first formulation to test for each client based on the learning models. One additional input point per iteration was added to each client’s data set to determine the next formulation to test.

The samples were formulated sequentially adding the M-alginate and CMCS precursor solutions followed by DIW in a 96-well plate. The sol mixture was then homogenized using a digital plate shaker (LSE digital microplate shaker; Corning) at 700 rpm for 30 min. AcOH was then added to make the total mass of each sample identical to those of the initial sample libraries, and the samples were mixed for 5 min at 700 rpm. The samples were then cross-linked as described in Section 2.3. Following hydrogel formation, the samples were characterized as described in Section 2.4, resulting in new labeled data. The newly labeled data were then incorporated into the learning models as described in Section 2.1, which generated the next-to-test formulations for all clients for the subsequent iteration. This process was repeated until an imposed budget was met.

### 2.6 Statistical Analysis

The reported results correspond to the mean and standard deviation of all measurements obtained from triplicate samples. A Student’s *t* test was used to characterize the significance level of the difference between the optimal target property obtained using collaborative vs independent learning.

## 3 Results and Discussion

### 3.1 Initializing Scalable Self-Driving AMD via AE and Collaborative Learning Using Labeled Data Acquired by HTE

**Figure 1:**
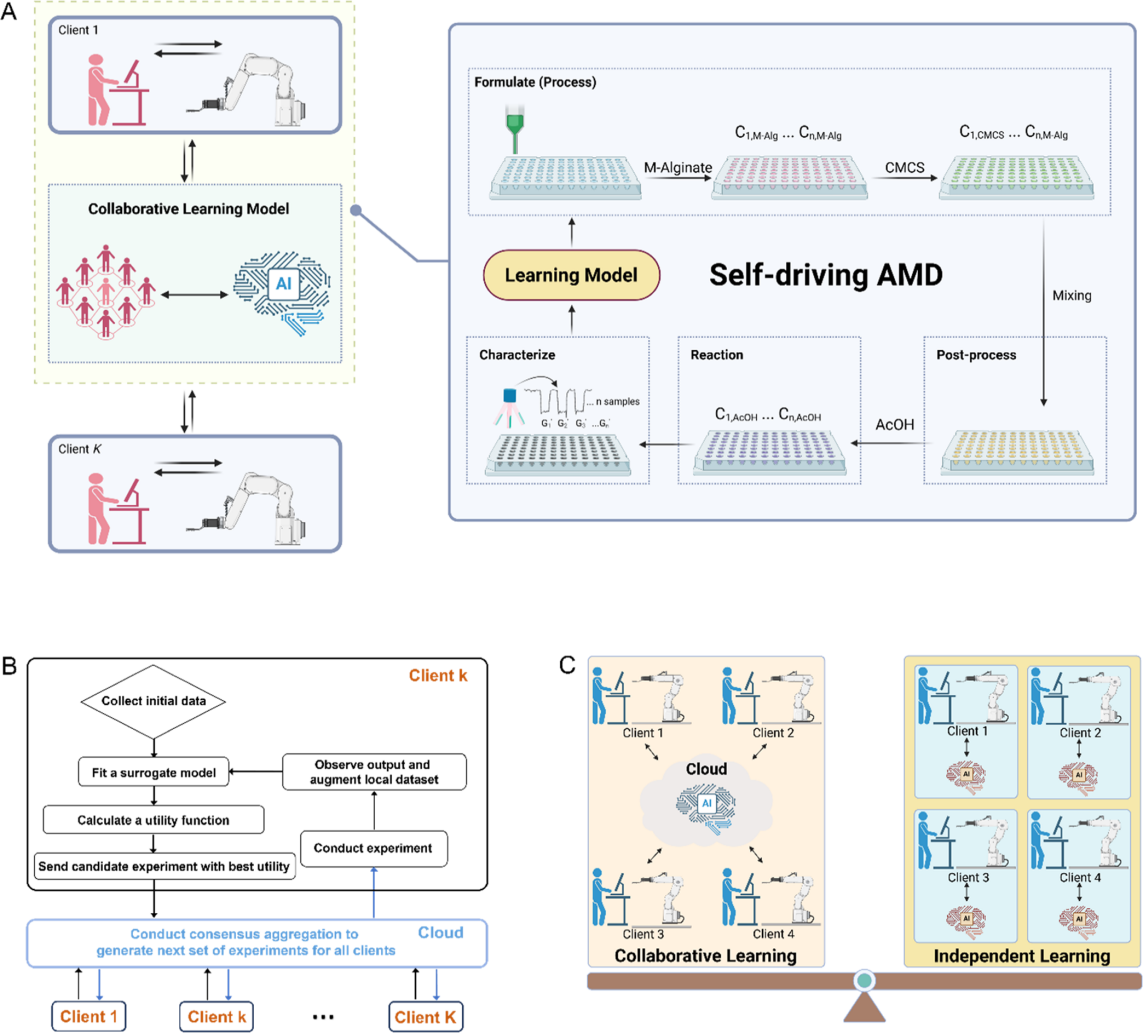
(A) Illustration of the scalable accelerated materials discovery (AMD) workflow driven by autonomous experimentation (AE) and collaborative learning. A novel collaborative learning model guides the selection of formulations to be tested by collaborating clients using labeled data generated by high-throughput experimentation (HTE). (B) Flowchart for scalable AMD via AE and collaborative learning based on consensus BO using a network of clients. (C) Illustration of the difference between traditional non-collaborative BO (i.e., independent learning) and collaborative BO with consensus (i.e., collaborative learning). Created in BioRender. Liu, Y. (2024) https://BioRender.com/r26y741.

As shown in Figure 1A, this work aimed to generate a scalable self-driving AMD workflow founded on AE and collaborative learning. We focus on a glycomaterial engineering application, given the opportunities associated with sustainability and the design challenges posed by the complexity of polysaccharide chemistry. Alginate and chitosan are important renewable glycomaterials that are widely used as scaffolds for tissue engineering and drug release applications.^39−44^ Alginate exhibits biocompatibility and tunable mechanical properties by controlling polymer content and cross-linking chemistry.^45^ Chitosan offers attractive mechanical properties and reactive groups, such as for applications requiring robust mechanical properties and chemical alteration.^46^ M-alginate is a multi-reducing end polysaccharide and forms hydrogels with CMCS. As shown in Figure 1B,C, we focus on a shared design objective among four clients who may work independently or collaboratively to discover the optimal formulation that maximizes the hydrogel mechanical properties, specifically *G′*, through a scaled AMD workflow.

**Figure 2:**
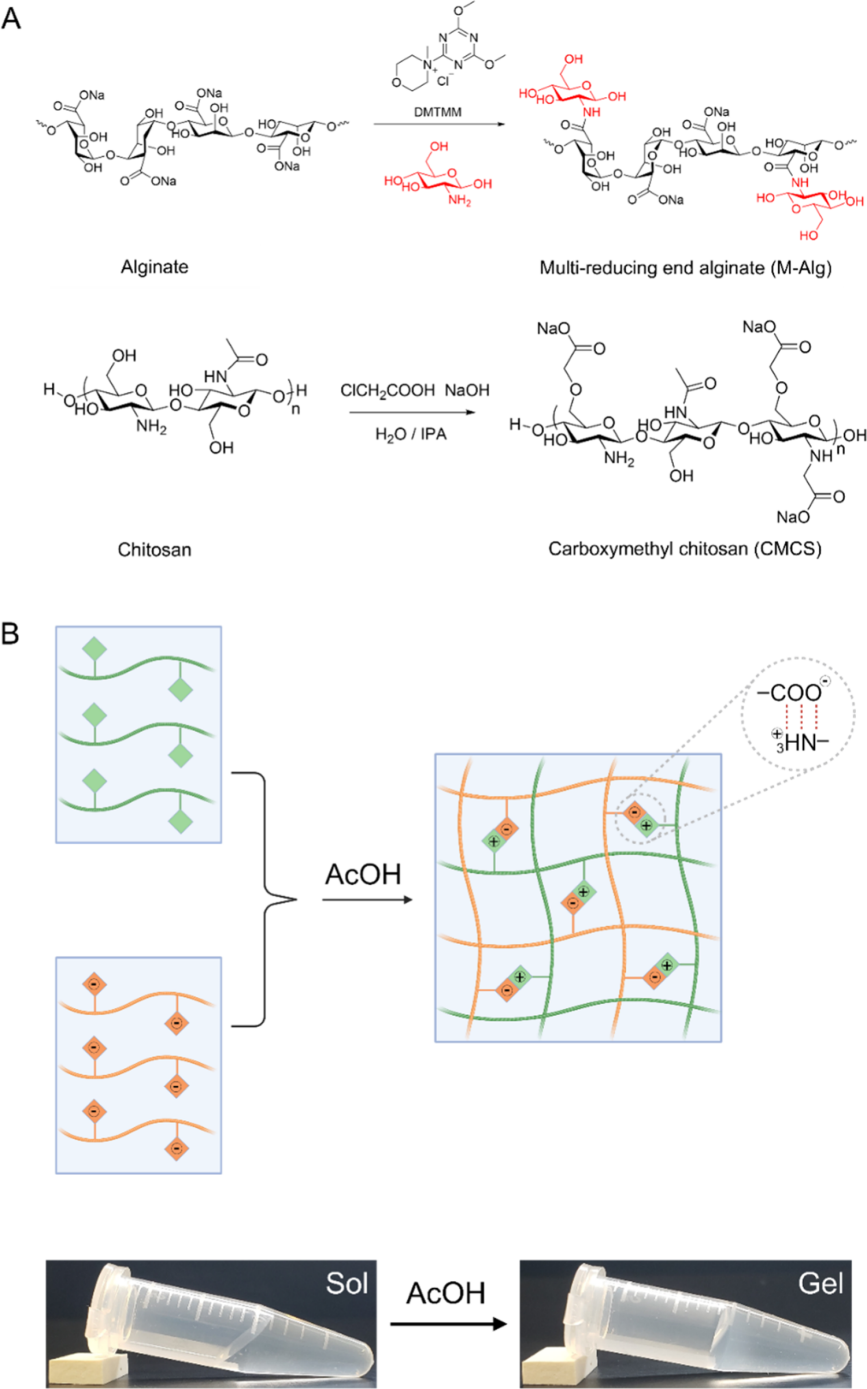
(A) Reaction schemes for synthesis of multi-reducing end alginate (M-alginate) and carboxymethyl chitosan sodium salt (CMCS). (B) Illustration and photographs of acid-catalyzed composite polysaccharide hydrogel cross-linking (gelation) in the presence of acetic acid (AcOH) (photographs: left = sol; right = gel; sample composition: M-alginate 0.37 wt %, CMCS 0.37 wt %, AcOH 1.72 wt %, DIW 97.54 wt %).

The reaction schemes to synthesize M-alginate and CMCS are shown in Figure 2A. While aqueous solutions of M-alginate and CMCS form hydrogels (see Figure 2B), it remains a challenge to discover optimal composite polysaccharide hydrogels that best combine the advantages of the individual polysaccharides due to the large formulation space and challenges of blending glycomaterials. In addition, the currently unknown gelation mechanism of M-alginate-CMCS hydrogels further motivates the use of HTE or AE to interrogate the material design space.

While considerable strides have been made in AMD applications to hard and brittle materials research, relatively less progress has been made in soft materials research applications, such as hydrogels, due to the challenges of adapting conventional methods for formulation, synthesis, and characterization of soft materials to autonomous or high-throughput formats^47,48^ and the high dimensionality of the material design spaces. For example, while solutions of M-alginate and CMCS can form composite hydrogels in the presence of AcOH (see Figure 2B),^49,50^ the resultant mechanical properties are highly dependent on the material formulation (i.e., four-component mixture), which contains M-alginate, CMCS, AcOH, and water. Thus, acid-catalyzed composite M-alginate-CMCS hydrogels exhibit a complex design space, which presents a challenge to AMD using brute-force HTE.

**Figure 3:**
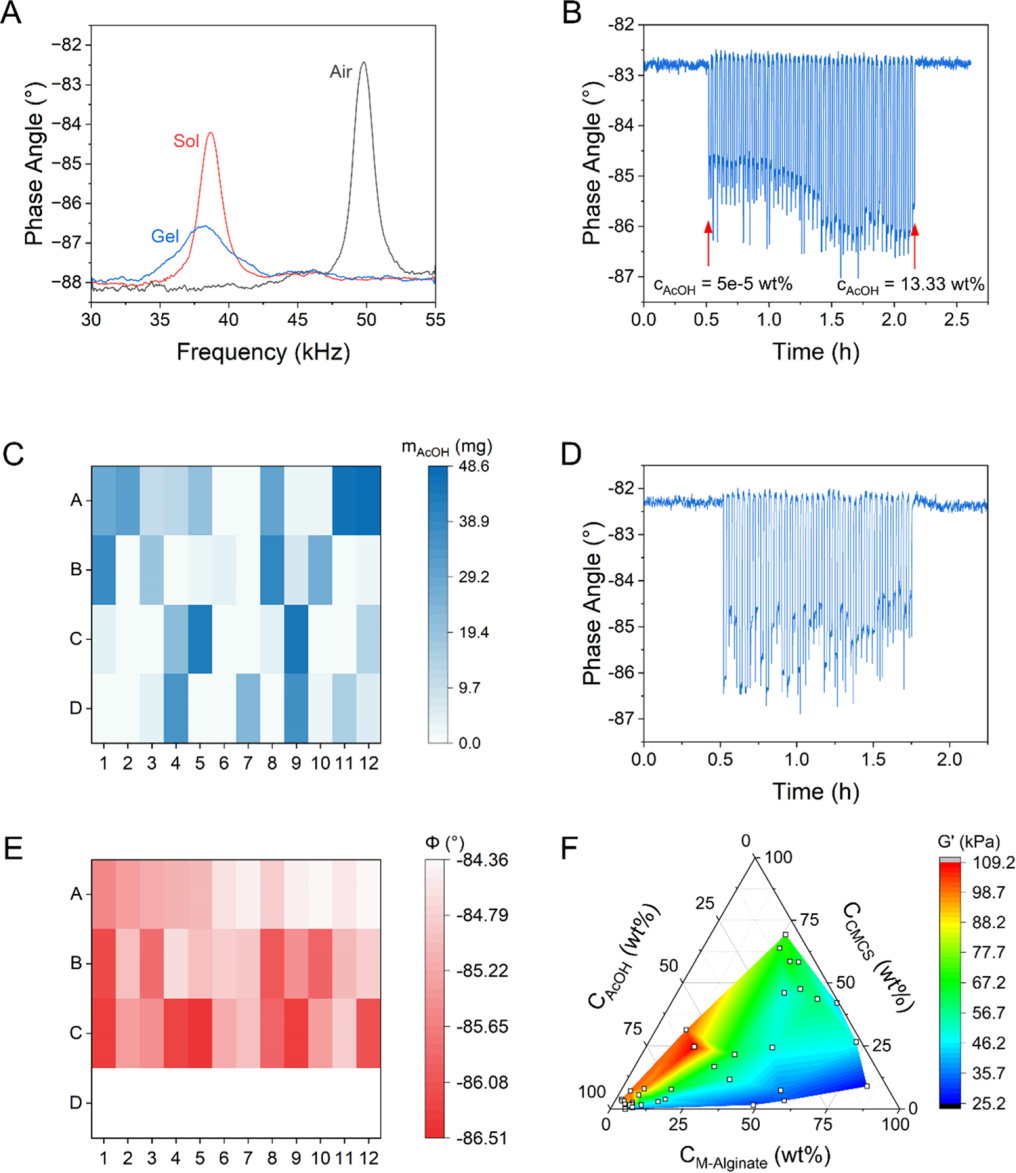
(A) Cantilever rheometer impedance spectra: (1) before measurement (air), (2) upon submersion in an M-alginate-CMCS precursor solution (sol), and (3) after gelation by reaction with AcOH for 12 h (gel). (B) Results of the HTE study that established the constraints on AcOH concentration used for M-alginate-CMCS composite hydrogel cross-linking. (C) Heat map showing the amount of AcOH dispensed in each sample of an initial 48-sample M-alginate-CMCS composite hydrogel library. (D) Real-time phase angle at resonance (φ(*t*)) response (i.e., raw HTC data) for the library described in (C). (E) Heat map of the steady-state φ response for the library described in (C) (12 samples were removed (i.e., row D) due to observed phase separation; thus, row D in the heat map does not contain sample information). (F) The composition–property relation for the M-alginate-CMCS composite hydrogel associated with the data in (E) presented as a ternary diagram (the DIW content of each sample is provided as Supporting Information).

The scalable AMD workflow driven by AE and collaborative learning is shown in Figure 1, which consists of an AE workflow for material synthesis and characterization driven by a group of clients with a shared material design objective. Representative impedance spectra of the cantilever rheometer are shown in Figure 3A before and after gelation for a single formulation. As shown in Figure S1, while the spectra of three different representative formulations before gelation were similar, although the mixtures exhibited significantly different compositions (sol 1: 0.25 wt % M-alginate and 0.41 wt % CMCS; sol 2: 0.29 wt % M-alginate and 0.37 wt % CMCS; sol 3: 0.42 wt % M-alginate and 0.24 wt %, CMCS), the spectra after gelation by AcOH addition (sol 1: 5.19 wt % AcOH; sol 2: 7.74 wt % AcOH; sol 3: 6.24 wt % AcOH) were significantly different. This result demonstrates the impact of the formulation on the composite hydrogel *G′*.

Given the complex design space associated with the composition–property relation of the acid-catalyzed composite M-alginate-CMCS hydrogel, we first examined the dependence of the sol–gel transition on the AcOH concentration by HTE using a 48-sample library in which the polymer content of all samples was constant (0.33 wt % M-alginate and 0.33 wt % CMCS). The total mass of each sample was 390 mg. As shown in Figure 3B, the relationship between φ and the AcOH concentration exhibited a sigmoidal trend. These data provided the upper and lower bounds (i.e., constraints) of AcOH content for the design space (48 and 0.02 mg per well equivalent to 12.31 and ∼5 × 10^–5^ wt %, respectively).

We next determined the design space constraints for the M-alginate and CMCS components by HTE using a second 48-sample library in which all samples exhibited an identical total polymer mass of 0.66 wt % (i.e., wt %_M-alginate_ + wt %_CMCS_ = 0.66 wt %) but the ratio of the macromolecular components was varied (see Figure 3C). Similarly, the total mass of each sample was 390 mg. The first and last samples in the library exhibited the lowest and highest relative amounts of M-alginate, respectively. The first and last samples in the library exhibited the highest and lowest relative amounts of CMCS, respectively. The AcOH content was randomly varied within the previously determined range to maximize the explored design space. As shown in Figure S2, the first 12 samples (i.e., composite polysaccharide hydrogels), which were rich in CMCS, exhibited phase separation and inhomogeneity. Thus, these compositions were removed from the design space. Figure 3D shows the raw HTC data for the other 36 samples, which formed homogeneous hydrogels. The corresponding steady-state value of φ for the 36 samples obtained by HTC is shown as a heat map in Figure 3E. The random distribution of φ across the material library and the absence of a sigmoidal trend observed in Figure 3B were expected results caused by the addition of AcOH in random volumes across the library. The corresponding composition–property relation data for the acid-catalyzed composite M-alginate-CMCS hydrogel is shown in Figure 3F as a ternary diagram and highlights the hydrogel *G*′ dependence on the formulation, specifically the AcOH content. We note that while the DIW content of each sample is not shown in Figure 3F, given the challenge of visualizing the composition–property relation for the four-component mixture, the DIW content of each sample can be obtained from knowledge of the total sample mass, which was identical for all samples. The DIW content of all samples shown in Figure 3F is provided as Supporting Information (see Supporting Data Set).

Relatively more labeled data were generated near the lower left corner of the diagram by design, given that the mass fraction of AcOH, which exhibited a maximum of 12.13 wt %, was higher than that of the polymer components, which exhibited a maximum of 0.66 wt %. The data in Figure 3F suggest that an imbalance in the mixture’s two macromolecular components resulted in relatively weak hydrogels. Increasing the AcOH mass fraction appeared to increase the composite hydrogel *G*′ regardless of the M-alginate:CMCS ratio. In summary, the two initial HTE studies generated the following material design constraints for the learning algorithms: 0.16 < wt %_M-Alginate_ < 0.66, 0.01 < wt %_CMCS_ < 0.45, 0.01 < wt %_AcOH_ < 10, and wt %_M-Alginate_ + wt %_CMCS_ = 0.66.

### 3.2 Scalable AMD via AE and Collaborative Learning with Consensus BO

Although brute-force HTE provides a methodology for exploring the design space and offers the potential for material discovery and optimization, it may be prohibitive in many applications from a resource expenditure perspective (i.e., physical or economic resource constraints), such as glycomaterial engineering applications. Also, there is no assurance that the formulations that exhibit promising performance or quality metrics are optimal. Alternatively, we assert that AE, in combination with collaborative learning, can establish a scalable self-driving AMD workflow capable of efficiently discovering an optimal design (i.e., formulation) within practical budget constraints. Thus, we used the labeled data generated by the initial HTE studies and the generated design constraints to efficiently explore the material design space by collaborative learning with consensus BO.

As shown in the left panel of Figure 1C, the methodology was validated by considering four clients that share an AMD objective, specifically, the aim to rapidly optimize *G*′ of an acid-catalyzed composite polysaccharide hydrogel composed of M-alginate and CMCS subject to an imposed budget. As shown in the right panel of Figure 1C, a control group was also examined based on independent learning (i.e., traditional BO) in which clients did not collaborate. Thus, for each iteration of the collaborative AMD workflow, the consensus BO model generated four different formulations to test (i.e., one per client). For example, given that all samples have identical total mass the four-component hydrogel can be framed as a three-component material design problem. Thus, *D* = 3 given the identical mass of all samples removes one degree of freedom from the material design problem and *K* = 4 given we consider a network of four collaborating clients. If we consider clients use the following consensus matrix
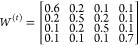
then, if the local utility maximizers of the clients are *x*_C_^(*t*)^ = ([1, 2, 1], [1, 2, 1], [2, 3, 3], [2, 1, 3])^*T*^, we obtain (*W*^(*t*)^ ⊗ *I*)*x*_*C*_^(*t*)^ = (1.2, 2, 1.4, 1.3, 2.1, 1.6, 1.5, 2.2, 2.1, 1.8, 1.4, 2.6), where *x*_1_^(*t*)new^ = (1.2, 2, 1.4)^*T*^ for client 1, *x*_2_^(*t*)new^ = (1.3, 2.1, 1.6)^*T*^ for client 2, *x*_3_^(*t*)new^ = (1.5, 2.2, 2.1)^*T*^ for client 3, and *x*_4_^(*t*)new^ = (1.8, 1.4, 2.6)^*T*^ for client 4. The independent learning model also contained four clients, each testing one formulation per iteration (we remind the reader that this group considers *W* = *I* in the consensus BO model).

To generate each client’s first formulation to test, we randomly sampled 20 of 36 labeled data points in Figure 3F without replacement and assigned them randomly to the four clients in each group. The learning models were initialized using five points per client since BO, and active learning in general, relies on a surrogate (a GP) to help inform the next data point to obtain, and such surrogates need at least three to six data points. Each client tested their formulation in triplicate to establish the error of the data label (i.e., the variance of *G*′ for a given formulation). HTC data for a single iteration involving 24 samples (8 clients × 1 sample/client × 3 replicates = 24 samples with eight different compositions, one unique composition per client), is shown in Figure S3 as a representative case. In many practical AMD applications, resource constraints limit the number of possible experimental iterations. Our budget constraints in this study restricted the number of iterations to eight. The number of data points used by each client and their respective learning model increased by one after each iteration, regardless of the group. Thus, consensus does not increase the number of data points used by the collaborative group relative to the control group; instead, it helps the collaborative group choose the next experiment better. We designed consensus in this way for two fundamental reasons: (1) privacy: sharing responses across clients may not respect privacy (e.g., sharing outcomes of medical treatment), and (2) heterogeneity: if clients have heterogeneity, then one client’s input–output tuple cannot be used in another client’s surrogate, as their BB functions exhibit differences.

### 3.3 Comparison of Scalable Self-Driving AMD Performance via Collaborative vs Independent AE

**Figure 4:**
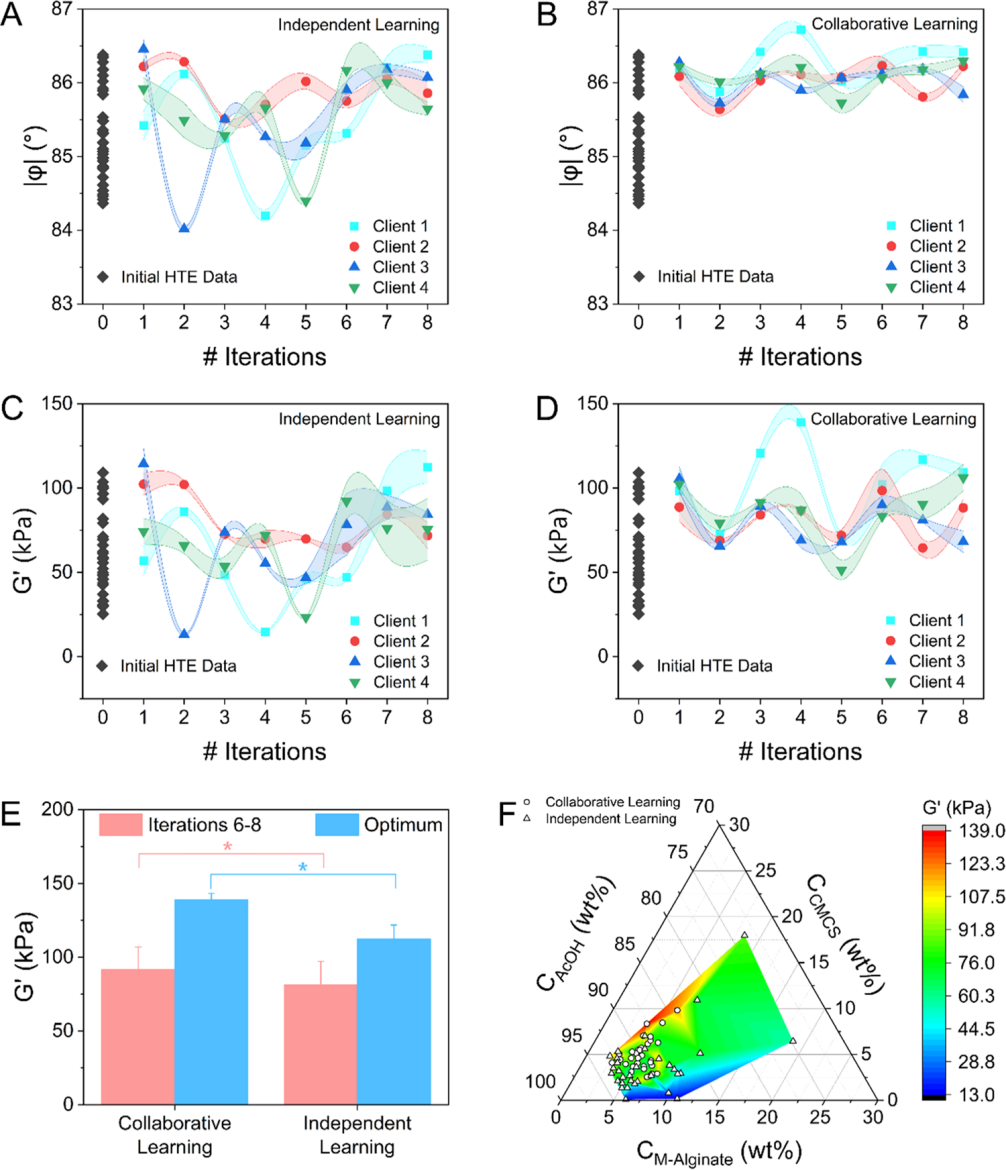
Absolute phase angle at resonance (|φ|) (A, B) and *G*′ (C, D) for scalable AMD driven by AE and collaborative learning with consensus BO vs traditional active learning by BO (i.e., independent learning). Labeled data acquired using brute-force HTE were used to inform the selection of formulations for iteration 0. (E) The mean *G*′ from iteration 6 to 8 and the optimal *G*′ obtained from collaborative learning and independent learning (* indicates *p* < 0.05). (F) Composition–property (*G*′) relation for the composite hydrogel generated by collaborative vs independent learning in terms of a ternary diagram (the DIW content of each sample is provided as Supporting Information).

The results of the collaborative learning-driven AMD workflow over eight iterations are shown in Figure 4 compared to the control group. Figure 4 displays the progress of the collaborative workflow in terms of the cantilever rheometer raw data and the associated *G*′. For client 1, the trend is increasing from iterations 1 to 4 (see Figure 4B,D). After iteration 4, the consensus BO algorithm exploits the current best solution or explores other promising regions, reflecting the exploration–exploitation nature of BO. This explains why the trend does not continue to increase steadily. The overall trend indicates that the consensus BO model has discovered a desirable solution after four iterations. Similarly, the overall trend fluctuates for other clients but generally increases, with the best result also discovered after several iterations. Despite the limited number of iterations, which we remind the reader is determined by the imposed budget limit, collaboration among four clients working toward the shared material design objective achieved superior performance relative to the non-collaborative clients within six iterations (see Figure 4A,C). As shown in Figure 4E, the formulations identified by the four collaborating clients across iterations 6–8 exhibited an average absolute phase angle value (|φ|) of 86.17 ± 0.18° and an average *G*′ of 91.6 ± 15.3 kPa. The average changes in average |φ| and *G*′ for successive iterations (across iterations 6–8) were 0.25 ± 0.18° and 27.1 ± 15.3 kPa, respectively. In contrast, the average |φ| and *G*′ across iterations 6–8 in the independent learning group were relatively smaller and exhibited larger variance (85.96 ± 0.28° and 81.2 ± 15.9 kPa, respectively). Likewise, the average changes in average |φ| and *G*′ for successive iterations (across iterations 6–8) and the associated variances were relatively larger in the control group (0.42 ± 0.28° and 34.0 ± 15.9 kPa, respectively). Figure 4E also shows a comparison of the mechanical properties associated with the optimal formulation identified by collaborative vs independent learning. The optimal formulation was identified in iteration 4 by client 1 in the collaborative learning group and iteration 8 by client 1 in the independent learning group.

Figure 4 demonstrates that the scaled, self-driving AMD workflow involving collaborating clients exhibited a more consistent trend in the target property to be optimized (*G*′) compared to the group that contained the same number of non-collaborating clients. We remind the reader that the storage modulus *E*′ can be obtained from *G*′ and the Poisson ratio, typically assumed to be 0.5 in hydrogels.^51^ In conclusion, the higher average *G*′ and *E*′ obtained by cooperating clients (*p* = 0.014) in the final budgeted iterations indicates the AMD scalability and improved performance offered by collaborative learning. Given the time required for each iteration was the same in both groups, the collaborative learning method also increased the efficiency of the scaled AMD workflow.

The trends in formulation discovery visualized as a composition–property relation are highlighted in Figure 4F for both groups. The DIW content of all samples is provided in Supporting Information. The ternary diagram highlights the data distribution that the collaborating and non-collaborating clients labeled through their search for an optimal formulation that produces a maximum hydrogel *G*′ and *E*′ (the initial labeled data obtained by brute-force HTE are excluded for clarity of data visualization). The collaborative learning model incentivized the cooperating clients to focus on exploration in the design space where *G*′ was relatively high. In contrast, the independent learning model drove the noninteracting clients to explore a larger design space where *G*′ exhibited a wide range. This is an expected result based on the design of the consensus BO model. For example, the consensus matrix (see eqs 5 and 6) is designed to support clients during the early stages when they lack sufficient data to fit high-quality surrogates, allowing them to leverage information from each other. In the later stages (i.e., iterations), we reduced the influence of different clients by shrinking the off-diagonal elements in the consensus matrix to enable clients to focus on their individual experiments and obtain client-specific optimal formulations.

While the insight into the physiochemical origins (e.g., bonding and structure) that describe a material’s given profile of physical or material properties is valuable, a detailed investigation of the composition–structure–property relation remains challenging. For example, given the complexity of polysaccharide chemistry, such as arising from the multi-reducing end nature of M-alginate used in this study, a detailed investigation of material structure is nontrivial and beyond the scope of this work. Hydrogels are bulk materials in which the local structures and interactions may vary. For example, the local structure inside polymer networks exhibits various potential forms, such as strands, physical entanglement, and chemical cross-linking.^52^

Despite its success in this case study, the current collaborative learning model has some limitations. First, the current model is limited to applications that involve single-objective optimization problems. Our ongoing work focuses on learning strategies for multiobjective optimization applications, such as the accelerated discovery of materials with several optimized performance and quality characteristics. Second, while applying the methodology to a three-dimensional design space is meaningful and establishes proof of principle, higher dimensionality is necessary for many other applications. For example, optimization of material processing parameters can also be included in the input space, given their potential impact on material structure and properties. Finally, the current collaborative learning model lacks explainability. For example, while adaptive sampling strategies can identify optimal formulations, additional work is required postdiscovery to understand the underlying chemistry and engineering principles that yield the process–composition–structure–property relation. Additional constraints guided by domain knowledge in chemistry and engineering can also potentially be integrated with the model to improve performance and explainability.

## 4 Conclusions

This study addressed a grand challenge in AE and AMD: scalability. While brute-force HTE can accomplish AMD for some materials, such as those with relatively simple design spaces and large budgets, the designer or manufacturer lacks certainty (e.g., quantitative assurance) that the identified formulation is optimal in the given design space, which is particularly challenging for complex functional materials, and an imposed budget will be optimally expended. We first demonstrated that self-driving AMD workflows can be scaled by AE and collaborative learning (i.e., “collaborative AE”), which distributes effort across multiple clients working toward a shared material design optimization objective. We showed that collaborative learning via consensus BO, which is based on cooperation among multiple clients, can establish a scalable self-driving AMD workflow that outperforms that driven by traditional independent learning using BO. The methodology was applied to AMD of novel composite polysaccharide hydrogels that exhibit optimal mechanical properties, specifically *G*′ and *E*′. This work demonstrates the capability of using consensus BO to scale AMD workflows using groups of interacting humans and machines.

## Data Availability

An open-source software implementation of consensus Bayesian optimization is available at https://github.com/UMDataScienceLab/Consensus_Bayesian_Opt/tree/main.

## Supporting Information Available

Additional methodological details of the fluid–structure interaction model; experimental results related to HTE studies, and a description of sample compositions, and Cantilever spectra; hydrogel photographs; raw sensor data, and summary of recent progress in literature (PDF)
Representative data set for AE (XLSX)
