# Supplementary material for: Scalable Accelerated Materials Discovery of Sustainable Polysaccharide-Based Hydrogels by Autonomous Experimentation and Collaborative Learning
Source: ACS Appl Mater Interfaces. 2024 Dec 11;16(51):70310–21. doi: 10.1021/acsami.4c16614 (PMC11672474; doi:10.1021/acsami.4c16614)
Supplement: Supplementary file 1 — am4c16614_si_001.pdf [file am4c16614_si_001.pdf]

Supporting Information

Scalable Accelerated Materials Discovery of Sustainable Polysaccharide-based Hydrogels by Autonomous Experimentation and Collaborative Learning

Yang Liu[1,2], Xubo Yue[3], Junru Zhang[1], Zhenghao Zhai[2], Ali Moammeri[1,4], Kevin J. Edgar[2, 5], Albert S. Berahas[6], Raed Al Kontar[6], Blake N. Johnson*[1,2,4,7]

[1] Grado Department of Industrial and Systems Engineering, Virginia Tech, Blacksburg, VA 24061, United States

[2] Macromolecules Innovation Institute, Virginia Tech, Blacksburg, VA 24061, United States

[3] Department of Mechanical and Industrial Engineering, Northeastern University, Boston, MA 02115, United States

[4] Department of Chemical Engineering, Virginia Tech, Blacksburg, VA 24061, United States

[5] Department of Sustainable Biomaterials, Virginia Tech, Blacksburg, VA 24061, United States

[6] Department of Industrial and Operations Engineering, University of Michigan, Ann Arbor, MI 48109, United States

[7] Department of Materials Science and Engineering, Virginia Tech, Blacksburg, VA 24061, United States

* Corresponding Author Contact Information:

Email: bnj@vt.edu; *Phone:* 540-231-0755; Fax: 540-231-3322

Address: 121 Durham Hall, 1145 Perry St, Blacksburg, VA 24061 United States

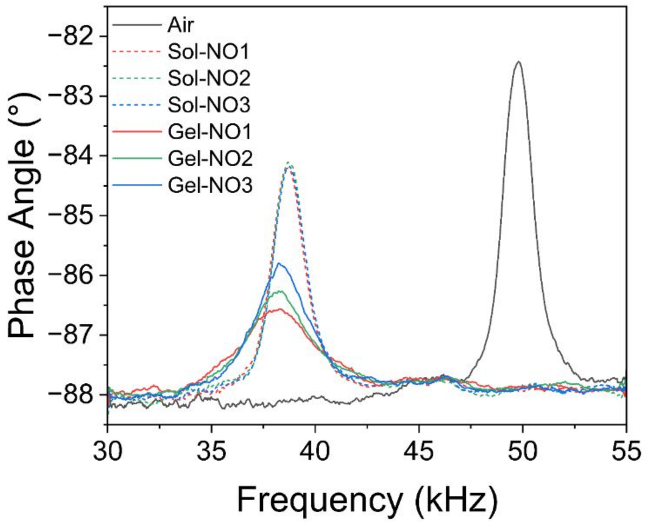

Figure S1. Spectra of the cantilever rheometer in three different M-alginate-CMCS mixtures (solutions) prior to AcOH addition and in the three resultant hydrogels illustrating the effect of mixture composition on hydrogel rheological properties (*G'*). A reference spectrum in air is also shown to illustrate the characteristics of the cantilever rheometer.

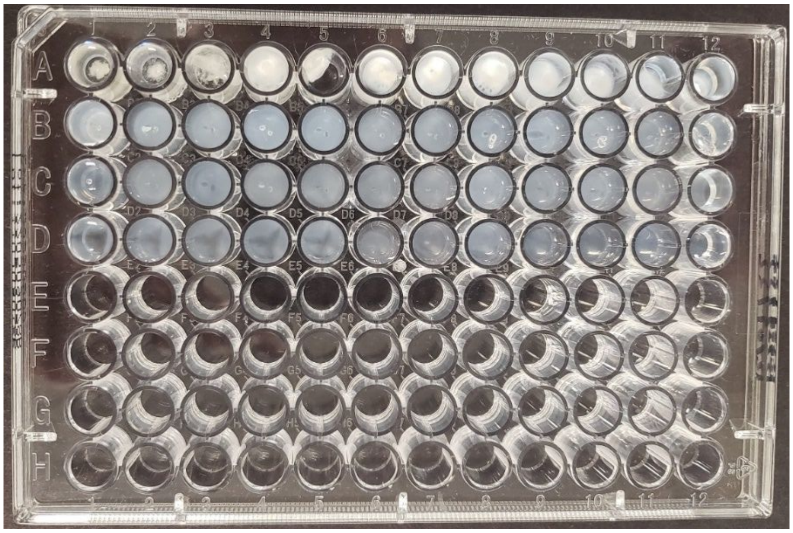

Figure S2. Photograph of a M-alginate-CMCS composite hydrogel library in a 96-well plate illustrating phase separation at high CMCS concentrations (see main text for well number descriptions).

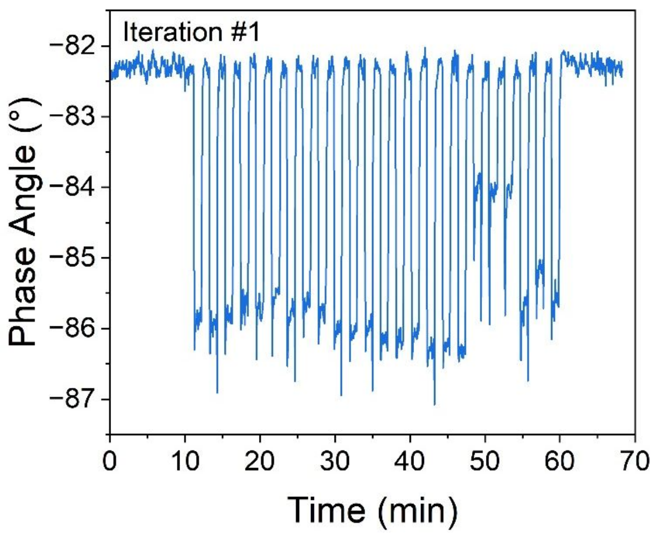

Figure S3. Representative time-series data of the cantilever rheometer from the well plate-based automated high-throughput characterization format for a single experimental iteration. Samples from eight clients are tested per iteration (four collaborating clients and four non-collaborating clients; each client examines three replicates).

Table S1. Highlight of recent progress in hydrogel applications of data-driven high-throughput experimentation. *Abbreviations:* HTS: High-throughput synthesis, HTC: High-throughput characterization, PEGMA: poly(ethylene glycol)methacrylate, SBMA: (2-(N-3-Sulfopropyl-N,N-dimethyl ammonium)ethyl methacrylate, PEGDMA: poly(ethylene glycol)dimethacrylate, PEG: poly(ethylene glycol), RGD: arginine-glycine-aspartic acid, HEMA: 2-hydroxyethyl methacrylate, DMAPS: 3-dimethyl-2-(2-methylprop-2-enoyloxy)ethyl azaniumyl propane-1-sulfonate, PEGDA: poly(ethylene glycol) diacrylate, FMA: 2,2,2-trifluoroethyl methacrylate, GelMA: Gelatin methacryloyl, P3HT: poly(3-hexylthiophene), PS: poly(styrene), PP: poly(propylene), PNIPAM: poly(N isopropylacrylamide), PEDOT: poly(3,4-ethylenedioxythiophene), PSS: polystyrene sulfonate, HA: Hyaluronic acid, RLGG: relative least general generalization, Fmoc-FF: N-fluorenylmethoxycarbonyl diphenylalanine, RF: Random Forest, NaSS: sodium p-styrenesulphonate, MPTC: 3-(methacryloylamino)propyl-trimethylammonium chloride, PVA: Poly(vinylalcohol), DNN: Deep neural network, GB: Gradient boosting, ANN: Artificial neural network, LR: logistic regression, NN: Nearest neighbor, SVM: support vector machine, BO: Bayesian optimization, CNFs: cellulose nanofibers, PAA: Poly(acrylic acid), XGBoost: extreme gradient boosting, DT: decision tree, ADA-GEL: Alginate Dialdehyde-Gelatin, γ-CD: γ-cyclodextrin, MLR: Multivariate linear regression.

Materials	Properties	HTS	HTC	Machine Learning	Ref.
PEGMA/SBMA/PEGDMA	Degradability	Microarray	Microscopic imaging	-	1
PEG/RGD	Cell culture	Microarray	Microscopic imaging	-	2
PEGDMA/HEMA/SBMA	Swelling, Drug loading	Microarray	Microscopic imaging	-	3
DMAPS/PEGDA/FMA/ Surfactants	Mechanical properties, Self-healing	Microfluidics	-	-	4
Gellan Gum/GelMA	Cell culture	Gradient	Microscopic imaging	-	5
P3HT/PS, PP/PS	Composition, Residence time	Gradient	Pulse tracer experiments	-	6

	distribution				
13 precursor polymers	Swelling, Compressive modulus, Degradation, Drug Release	Well-plate-based automated dispensing	Robot-assisted indentation	-	7
PEGDMA, Alginate/PNIPAM, PEGDMA/PEDOT:PSS	Rheology, Resistivity	Well-plate-based automated dispensing	Robot-assisted cantilever sensor	-	8
Collagen/Fibrinogen/HA	Rheology, Printability	-	-	RLGG	9
HA/Carbopol 980 NF/ Laponite XLG/Fmoc-FF	Rheology, Printability	-	-	RF	10
P(NaSS-co-MPTC), PVA	Viscoelasticity	-	-	DNN	11
Alginate	Rheology, Injectability	-	-	RF, GB	12
GelMA	Stiffness, Gelation time	-	-	ANN	13
Tetrapeptides	Hydrogel-forming ability	-	-	RF, LR, NN, SVM	14
Polyacrylamide/Alginate	Elongation, Fracture energy, Hysteresis effect, Resistivity, Strain sensitivity	-	-	BO	15
CNFs/PAA	Adsorption of toxic metals	-	-	XGBoost, BO	16
71 Nucleoside derivatives	Hydrogel-forming ability	-	-	XGBoost, LR, DT, RF	17
ADA-GEL	Stiffness	-	-	XGBoost	18
23 monomers and 4 crosslinkers	Photodegradation	Microarray	Microscopic imaging	BO	19
PEG/γ-CD	Compressive moduli, Compressive toughness, Mechanical hysteresis	Well-Plate-based	-	RF, MLR	20
Collagen, Pluronic F127, Alginate/PNIPAM	Gelation	Well-plate-based	Robot-assisted cantilever sensor	SVM, RF, XGBoost	21

References

(1) Rosenfeld, A.; Oelschlaeger, C.; Thelen, R.; Heissler, S.; Levkin, P. A. Miniaturized high-throughput synthesis and screening of responsive hydrogels using nanoliter compartments. *Mater. Today Bio* **2020**, *6*, 100053.

(2) Hao, H.; Huang, J.; Liu, P.; Xue, Y.; Wang, J.; Ren, K.; Jin, Q.; Ji, J.; Greiner, A.; Agarwal, S. Rapid build-up of high-throughput screening microarrays with biochemistry gradients via light-induced thiol–ene "click" chemistry. *J. Mater. Chem. B* **2021**, *9* (13), 3032-3037.

(3) Yang, J.; Ran, Y.; Huang, L.; Ren, C.; Hao, X.; Ma, L.; Zhang, D. High-throughput screening of zwitterion-based coatings towards improved mechanical stability and drug-loading capacity. *npj Mater. Degrad.* **2023**, *7* (1), 51.

(4) Ding, Y.; Tang, H.; Zhang, C.; Li, W.; Li, G.; Zhang, Y.; Xu, C.; Zhao, F.; Guo, Q.; Guo, C. F.; et al. High-Throughput Screening of Self-Healable Polysulfobetaine Hydrogels and their Applications in Flexible Electronics. *Adv. Funct. Mater.* **2021**, *31* (18), 2100489.

(5) Guimarães, C. F.; Gasperini, L.; Ribeiro, R. S.; Carvalho, A. F.; Marques, A. P.; Reis, R. L. High-throughput fabrication of cell-laden 3D biomaterial gradients. *Mater. Horiz.* **2020**, *7* (9), 2414-2421.

(6) Liu, A. L.; Dogan-Guner, E. M.; McBride, M.; Venkatesh, R.; Gonzalez, M. A.; Reichmanis, E.; Grover, M.; Meredith, J. C. Composition Gradient High-Throughput Polymer Libraries Enabled by Passive Mixing and Elevated Temperature Operability. *Chem. Mater.* **2022**, *34* (15), 6659-6670.

(7) Xu, F.; Corbett, B.; Bell, S.; Zhang, C.; Budi Hartono, M.; Farsangi, Z. J.; MacGregor, J.; Hoare, T. High-Throughput Synthesis, Analysis, and Optimization of Injectable Hydrogels for Protein Delivery. *Biomacromolecules* **2020**, *21* (1), 214-229.

(8) Liu, Y.; Zhang, J.; Zhang, Y.; Yoon, H. Y.; Jia, X.; Roman, M.; Johnson, B. N. Accelerated Engineering of Optimized Functional Composite Hydrogels via High-Throughput Experimentation. *ACS Appl. Mater. Interfaces* **2023**, *15* (45), 52908-52920.

(9) Lee, J.; Oh, S. J.; An, S. H.; Kim, W. D.; Kim, S. H. Machine learning-based design strategy for 3D printable bioink: elastic modulus and yield stress determine printability. *Biofabrication* **2020**, *12* (3), 13.

(10) Nadernezhad, A.; Groll, J. Machine Learning Reveals a General Understanding of Printability in Formulations Based on Rheology Additives. *Adv. Sci.* **2022**, *9* (29), 11.

(11) Wang, J.; Zhu, B. G.; Hui, C. Y.; Zehnder, A. T. Determination of material parameters in constitutive models using adaptive neural network machine learning. *J. Mech. Phys. Solids* **2023**, *177*, 14.

(12) Verheyen, C. A.; Uzel, S. G. M.; Kurum, A.; Roche, E. T.; Lewis, J. A. Integrated data-driven modeling and experimental optimization of granular hydrogel matrices. *Matter* **2023**, *6* (3), 1015-1036.

(13) Karaoglu, I. C.; Kebabci, A. O.; Kizilel, S. Optimization of Gelatin Methacryloyl Hydrogel Properties through an Artificial Neural Network Model. *ACS Appl. Mater. Interfaces* **2023**, *15* (38), 44796-44808.

(14) Xu, T. Y.; Wang, J. Q.; Zhao, S.; Chen, D. H.; Zhang, H. Y.; Fang, Y.; Kong, N.; Zhou, Z.; Li, W. B.; Wang, H. M. Accelerating the prediction and discovery of peptide hydrogels with human-in-the-loop. *Nat. Commun.* **2023**, *14* (1), 12.

(15) Xu, S. H.; Chen, X.; Wang, S.; Chen, Z. W.; Pan, P. H.; Huang, Q. L. Integrating machine learning for the optimization of polyacrylamide/alginate hydrogel. *Regen. Biomater.* **2024**, *11*, 10.

(16) Zhang, J.; Fu, K. X.; Wang, D. W.; Zhou, S. Q.; Luo, J. M. Refining hydrogel-based sorbent design for efficient toxic metal removal using machine learning-Bayesian optimization. *J. Hazard. Mater.* **2024**, *479*, 11.

(17) Li, W. Q.; Wen, Y. H.; Wang, K. C.; Ding, Z. H.; Wang, L. F.; Chen, Q. M.; Xie, L.; Xu, H.; Zhao, H. Developing a machine learning model for accurate nucleoside hydrogels prediction based on descriptors. *Nat. Commun.* **2024**, *15* (1), 16.

(18) Ege, D.; Boccaccini, A. R. Investigating the Effect of Processing and Material Parameters of Alginate Dialdehyde-Gelatin (ADA-GEL)-Based Hydrogels on Stiffness by XGB Machine Learning Model. *Bioengineering-Basel* **2024**, *11* (5), 14.

(19) Seifermann, M.; Reiser, P.; Friederich, P.; Levkin, P. A. High-Throughput Synthesis and Machine Learning Assisted Design of Photodegradable Hydrogels. *Small Methods* **2023**, *7* (9), 11.

(20) Tang, M.; Zheng, D.; Samanta, J.; Tsai, E. H. R.; Qiu, H. B.; Read, J. A.; Ke, C. F. Reinforced double-threaded slide-ring networks for accelerated hydrogel discovery and 3D printing. *Chem* **2023**, *9* (12), 18.

(21) Zhang, J.; Liu, Y.; Chandra Sekhar.P, D.; Singh, M.; Tong, Y.; Kucukdeger, E.; Yoon, H. Y.; Haring, A. P.; Roman, M.; Kong, Z.; et al. Rapid, autonomous high-throughput characterization of hydrogel rheological properties via automated sensing and physics-guided machine learning. *Appl. Mater. Today* **2023**, *30*, 101720.
